# Peripheral Neuron Survival and Outgrowth on Graphene

**DOI:** 10.3389/fnins.2018.00001

**Published:** 2018-01-22

**Authors:** Domenica Convertino, Stefano Luin, Laura Marchetti, Camilla Coletti

**Affiliations:** ^1^NEST, Scuola Normale Superiore, Pisa, Italy; ^2^Center for Nanotechnology Innovation @NEST, Istituto Italiano di Tecnologia, Pisa, Italy

**Keywords:** graphene, neuron culture coating, peripheral DRG neuron, PC12, differentiation

## Abstract

Graphene displays properties that make it appealing for neuroregenerative medicine, yet its interaction with peripheral neurons has been scarcely investigated. Here, we culture on graphene two established models for peripheral neurons: PC12 cells and DRG primary neurons. We perform a nano-resolved analysis of polymeric coatings on graphene and combine optical microscopy and viability assays to assess the material cytocompatibility and influence on differentiation. We find that differentiated PC12 cells display a remarkably increased neurite length on graphene (up to 27%) with respect to controls. Notably, DRG primary neurons survive both on bare and coated graphene. They present dense axonal networks on coated graphene, while they form cell islets characterized by dense axonal bundles on uncoated graphene. These findings indicate that graphene holds potential for nerve tissue regeneration and might pave the road to novel concepts of active nerve conduits.

## Introduction

A specific feature of peripheral nerves is the ability to spontaneously regenerate after traumatic injuries. In the presence of important gaps where an end-to-end suture is not possible, a surgical approach is used, where nerve conduits (generally, autografts, or allografts) are used as bridges between the nerve stumps and provide physical guidance for the axons (Faroni et al., 2015). However, they present limitations in functional recovery and other disadvantages, e.g., size mismatch and increasing healing time for autografts, and rejection and disease transmission for allografts (Daly et al., 2012). A promising alternative is represented by tissue engineered nerve grafts, that have shown to improve regeneration, reduce scar formation and increase the concentration of neurotrophic factors (Gu et al., 2014; Faroni et al., 2015). Among materials that can be used for the guide production, silicon stimulates excessive scar tissue formation thus lacking long-term stability, while some other natural polymers, such as collagen and chitosan, lack adequate mechanical and electrical properties (Tran et al., 2009; Fraczek-Szczypta, 2014; Pinho et al., 2016). In recent years, new materials have been suggested as alternative candidates for tissue engineering applications. In particular graphene and other carbon-based nanomaterials have been proposed in life-science applications and nerve tissue regeneration (Fraczek-Szczypta, 2014; Kostarelos and Novoselov, 2014; Ding et al., 2015).

Graphene is a monolayer of sp2-hybridized carbon atoms arranged in a two-dimensional honeycomb lattice that was first isolated in 2004 from graphite (Novoselov et al., 2004). The increasing research interest in graphene is due to its incredible properties: high electron mobility (also at room temperature), superior mechanical properties both in flexibility and strength, high thermal conductivity and high area/volume ratio (Lee et al., 2008; Castro Neto et al., 2009). Furthermore, its biocompatibility and chemical stability make it ideally suited for biomedical applications (Bitounis et al., 2013).

Several studies have used graphene-based materials as biocompatible substrates for growth, differentiation and stimulation of different cell types, including neural cells (Agarwal et al., 2010; Li et al., 2011; Park et al., 2011; Bendali et al., 2013; Sahni et al., 2013; Tang et al., 2013; Bramini et al., 2016; Defterali et al., 2016; Fabbro et al., 2016; Guo et al., 2016; Rauti et al., 2016; Veliev et al., 2016). Polymer-coated graphene was shown to enhance the differentiation of neural stem cells (NSC) into neurons (Park et al., 2011), influencing their passive and active bioelectric properties (Tang et al., 2013; Guo et al., 2016). In addition, coated graphene-based materials were found to accelerate neurite sprouting and outgrowth of mouse hippocampal neurons (Li et al., 2011) and PC12 cells (Agarwal et al., 2010). A number of studies have also analyzed the effect of uncoated graphene-based materials on neural cells. Defterali et al. showed that uncoated thermally reduced graphene favored neural stem cells differentiation (Defterali et al., 2016). Neuron synapse formation and activity were not affected by graphene produced by liquid phase exfoliation (Fabbro et al., 2016), while an impairment of excitatory transmission was observed in primary neurons following a chronic exposure to graphene oxide flakes (Bramini et al., 2016; Rauti et al., 2016). Bare graphene was shown to be biocompatible, sustaining neuron survival and neurite outgrowth (Bendali et al., 2013; Sahni et al., 2013; Veliev et al., 2016), although the presence of defects may reduce the neural affinity, preventing cell attachment (Veliev et al., 2016). To date, most biomedical studies have investigated graphene covalent-functionalized forms such as graphene oxide (GO) and its chemical reduction known as reduced graphene oxide (RGO), or liquid phase exfoliated graphene (Agarwal et al., 2010; Bitounis et al., 2013; Bramini et al., 2016; Defterali et al., 2016; Fabbro et al., 2016; Rauti et al., 2016; Liu et al., 2017). These graphene-like structures have altered electronic structure and physical properties due to the variable fraction of sp2 and sp3 hybridized carbon atoms. With respect to those graphene-based materials, pristine graphene offers enhanced electrical and tribological properties and most notably an excellent electrical conductivity thus prospecting advantages for nervous system regeneration applications. Indeed, it has been demonstrated that conductive materials can enhance the electric field produced by the cell, influencing cell bioelectric properties (Guo et al., 2016). Electrical stimulation can also enhance and directs neurite outgrowth (Schmidt et al., 1997; Meng, 2014) and can accelerate axonal elongation (Fraczek-Szczypta, 2014). Neural conductive interfaces for neural regeneration application usually exploit conductive polymers, such as polyethylenedioxythiophene (PEDOT) and polypyrrole (PPy), or composite materials whose conductivity depends on the inclusion of graphene or carbon nanotubes (CNTs) (Schmidt et al., 1997; Deng et al., 2011; Pinho et al., 2016). Recently, graphene and carbon nanotubes (CNTs) have been successfully used to improve recording and electrical stimulation of neurons (Keefer et al., 2008; Kuzum et al., 2014) and surprisingly neural microelectrode arrays (MEAs) fabricated using graphene performed better than gold and indium tin oxide (ITO), in terms of signal-to-noise ratio (SNR) (Rastegar et al., 2017).

To date, the interaction between pristine graphene and peripheral neural cells has been investigated only in two studies (Lee et al., 2012; Hong et al., 2014), which suggest a positive effect on neurite outgrowth and proliferation when using graphene coated with fetal bovine serum (FBS). However, in both studies bare glass is used as control, thus the effect on the results of FBS coating, which *per se* is not a traditional coating for neural cells (Sun et al., 2012), is not investigated. No detailed study has yet examined the homogeneity and quality of the coatings typically adopted in neuronal culture. Predicting how polymeric surface coatings distribute onto graphene, due to its hydrophobicity and extreme flatness, is by no means trivial; furthermore, understanding how nerve cells can sense graphene under extracellular-matrix-like coatings is crucially important for possible *in vivo* applications. Overall, this lack of studies on pristine graphene leaves other carbon-based materials such as carbon nanofibers (CNF), carbon nanotubes (CNT), GO and rGO to star in its play (Ku et al., 2013; Fraczek-Szczypta, 2014; Ding et al., 2015; Liu et al., 2017).

In this work we investigate the potential of graphene as a conductive peripheral neural interface. We select epitaxial graphene obtained via thermal decomposition on silicon carbide (SiC) (Starke et al., 2012) as the ideal substrate for such investigations. In fact, epitaxial graphene on SiC combines high crystalline quality, scalability, thickness homogeneity and an extreme cleanliness. Graphene is used as a substrate for two cellular models: (i) PC12 cells, a non-neuronal cell line that is able to differentiate upon Nerve Growth Factor (NGF) stimulation and constitutes a widely-used model for peripheral sympathetic neurons (Greene and Tischler, 1982); (ii) dorsal root ganglion (DRG) sensory neurons, which are used as a model to study regenerative axon growth (Chierzi et al., 2005). The homogeneity and quality of a number of polymeric coatings typically adopted for neuronal culturing is investigated, and the most suitable ones are identified and adopted for the reported cultures. Furthermore, DRG neurons are also interfaced with bare graphene to assess their interaction with graphene *per se*, in the absence of a coating. Optical microscopy is used to investigate neurite length, number and differentiation, while viability assays are used to assess cytocompatibility. We compared results on monolayer graphene on SiC (G) with the ones on 4 possible control substrates: hydrogen etched SiC (SiC), gold coated glass coverslip (Au), glass coverslip (Glass) and polystyrene plate (well). The latter, being routinely used in cell culture procedures, was used as classic control. SiC controls were implemented since graphene was grown directly on such substrates, which display a good biocompatibility (Saddow et al., 2011) and present prospects for neural implants (Frewin et al., 2013). Finally, glass coverslips were coated with a very thin layer of gold to mimic the graphene layer grown on SiC. We used gold substrates as conductive controls, as gold, together with platinum (Pt, especially its porous form Pt-black), titanium nitride (TiN) and iridium oxide (IrOx), is typically interfaced with neurons in the fabrication of biomedical electrodes (Kim et al., 2014; Obien et al., 2015); Pt-Black, TiN, and IrOx are useful for the increased effective surface (Aregueta-Robles et al., 2014).

## Materials and methods

### Substrates preparation and characterization

Graphene on SiC was prepared by adopting a technique which allows to obtain quasi-free standing monolayer graphene (QFMLG) (Riedl et al., 2009). Briefly, buffer layer graphene was obtained via thermal decomposition of on-axis 4H-SiC(0001) performed at 1,250°C in argon atmosphere. QFMLG was obtained by hydrogen intercalating the buffer layer samples at 900°C in molecular hydrogen at atmospheric pressure (Bianco et al., 2015). The controls adopted in the experiments were: (i) Hydrogen etched SiC(0001) dices (the same substrates where graphene was grown) were cleaned with HF to remove the oxide layer, and hydrogen etched at a temperature of 1,250°C as previously reported (Frewin et al., 2009). (ii) Gold coated glass coverslips were obtained by thermally evaporating on the coverslips, previously cleaned with oxygen plasma, a 2 nm titanium adhesive layer and a 4 nm thin gold layer. (iii) Bare glass coverslips were treated overnight with 65% nitric acid (Sigma-Aldrich). (iv) Polystyrene 48-well plates (Corning). The dimensions of all the substrates were about 6 × 6 mm^2^. The topography of the samples as well as the graphene number of layers and quality were assessed by both AFM and Raman spectroscopy (Figure S1). Before cell culture, all substrates were sterilized by 30 min immersion in 96% ethanol and then rinsed several times with deionized (DI) water.

### Surfaces functionalization

Samples were coated with different polymeric solutions suggested for the targeted cell cultures and AFM analyses were performed to investigate the morphology of such coatings on graphene and the controls. The following solutions were tested: 100 μg/ml Poly-L-lysine (PLL) solution in water (Sigma-Aldrich), 200 μg/ml Collagene Type I (Sigma-Aldrich) in DI water, 30 μg/ml Poly-D-lysine (PDL) (Sigma-Aldrich) in PBS, 30 μg/ml PDL and 5 μg/ml laminin (Life Technologies) in PBS. The samples were incubated with the coating solution at 37°C for 1, 4, and 12 h and rinsed three times in DI water before analyzing their topography via AFM. AFM was performed in tapping mode on samples with and without the polymeric coating, over several areas up to 10 × 10 μm wide. AFM micrographs were analyzed using the software Gwyddion 2.45.

### PC12 cell culture

PC12 cells (ATCC® CRL-1721™) were maintained in a humidified atmosphere at 37°C, 5% CO2 in RPMI 1640 medium supplemented with 10% horse serum, 5% fetal bovine serum, 1% penicillin/streptomycin and 1% L-glutamine (Gibco). Cells were plated at ~40–60% confluency onto the substrates previously coated with 100 μg/ml Poly-L-lysine solution (PLL) in water (Sigma-Aldrich). Differentiation was achieved using two different procedures: (1) direct addition of 50 ng/ml NGF (Alomone Labs) in complete cell medium after seeding; (2) a 5–6 days priming with 15 ng/ml NGF in complete medium, followed by seeding on the substrates with 50 ng/ml NGF in RPMI medium supplemented with 1% horse serum, 0.5% fetal bovine serum, 1% penicillin/streptomycin and 1% L-glutamine. In both cases, 2/3 of the medium was renewed every 2–3 days. With the second procedure an improved differentiation was observed. The cells were observed at different time points using an inverted microscope equipped with a 20 × /40 × magnification objective (Leica DMI4000B microscope). Typically, 10 fields per sample were acquired to perform morphometric analysis of PC12 differentiation. Three parameters were measured as previously reported (Marchetti et al., 2014): (i) the percentage of differentiated cells (Diff), determined counting the number of cells with at least one neurite with a length equal to or longer than the cell body diameter; (ii) the average number of neurites per cell in the field (av. neurites/cell); (iii) the mean neurite length measuring the longest neurite of each differentiated cell in the field (length). The calculated values of Diff, Av. neurites/cell and Length are reported in **Figure 2**. Cell viability was assessed with the Cell counting Kit-8 assay (CCK-8, Sigma-Aldrich), based on quantification of WST reduction due to the metabolic activity of viable cells. Samples were prepared according to the manufacturer's instructions and measured at the GloMax® Discover multiplate reader (Promega). The results are reported as % over the polystyrene well, considered as control. All the experiments were repeated at least twice independently.

### DRG cell culture

Rat Embryonic Dorsal Root Ganglion Neurons (R-EDRG-515 AMP, Lonza) cells were maintained in a humidified atmosphere at 37°C, 5% CO2 in Primary Neuron Basal Medium (PNBM, Lonza) supplemented with L-glutamine, antibiotics and NSF-1 (at a final concentration of 2%) as recommended by the manufacturer. Neurons were plated on the substrates previously coated with a PBS solution of 30 μg/ml Poly-D-lysine (Sigma-Aldrich) (PDL) and 5 μg/ml laminin (Life Technologies). The medium was always supplemented with 100 ng/ml of NGF (Alomone Labs). Since 24 h after seeding, 25 μM AraC (Sigma-Aldrich) was added for inhibition of glia proliferation. Half of the medium was replaced every 3–4 days. Neurons were observed at different time points using an inverted microscope (Leica DMI4000B microscope).

### Statistical analysis

For all the experiments, we performed two independent cultures with two biological duplicates each. For the morphometric analysis of the PC12 cells, for each substrate we analyzed at least 200 cells (nc = number of cell) from selected fields (nf = number of field) of the four replicates (two biological duplicates per culture) obtained with a 40 × objective (Au: nf = 17, nc = 203; Glass: nf = 33, nc = 1,106; G: nf = 42, nc = 877; SiC: nf = 35, nc = 1,004; well: nf = 37, nc = 724). For the DRG neurons we analyzed nf fields using a 40 × objective for a total of nc cells for each substrate (day 1: Au, nf = 13, nc = 67, Glass: nf = 14, nc = 75; G: nf = 13, nc = 29; SiC: nf = 12, nc = 35; day 2: Au, nf = 16, nc = 89, Glass: nf = 13, nc = 100, G: nf = 12, nc = 34, SiC: nf = 11, nc = 37). The number of cells analyzed (nc) is the total pool of the four experiments. All data are expressed as the average value (mean) ± standard error of the mean (SE) unless stated otherwise. Data were analyzed by using Origin Software and nonparametric Kruskal–Wallis test with Dunn's multiple comparison test were used for statistical significance with ^*^p < 0.05, ^**^p < 0.01, and ^***^p < 0.001.

## Results and discussion

### Polymeric coating of epitaxial graphene and control substrates

**Figure 1:**
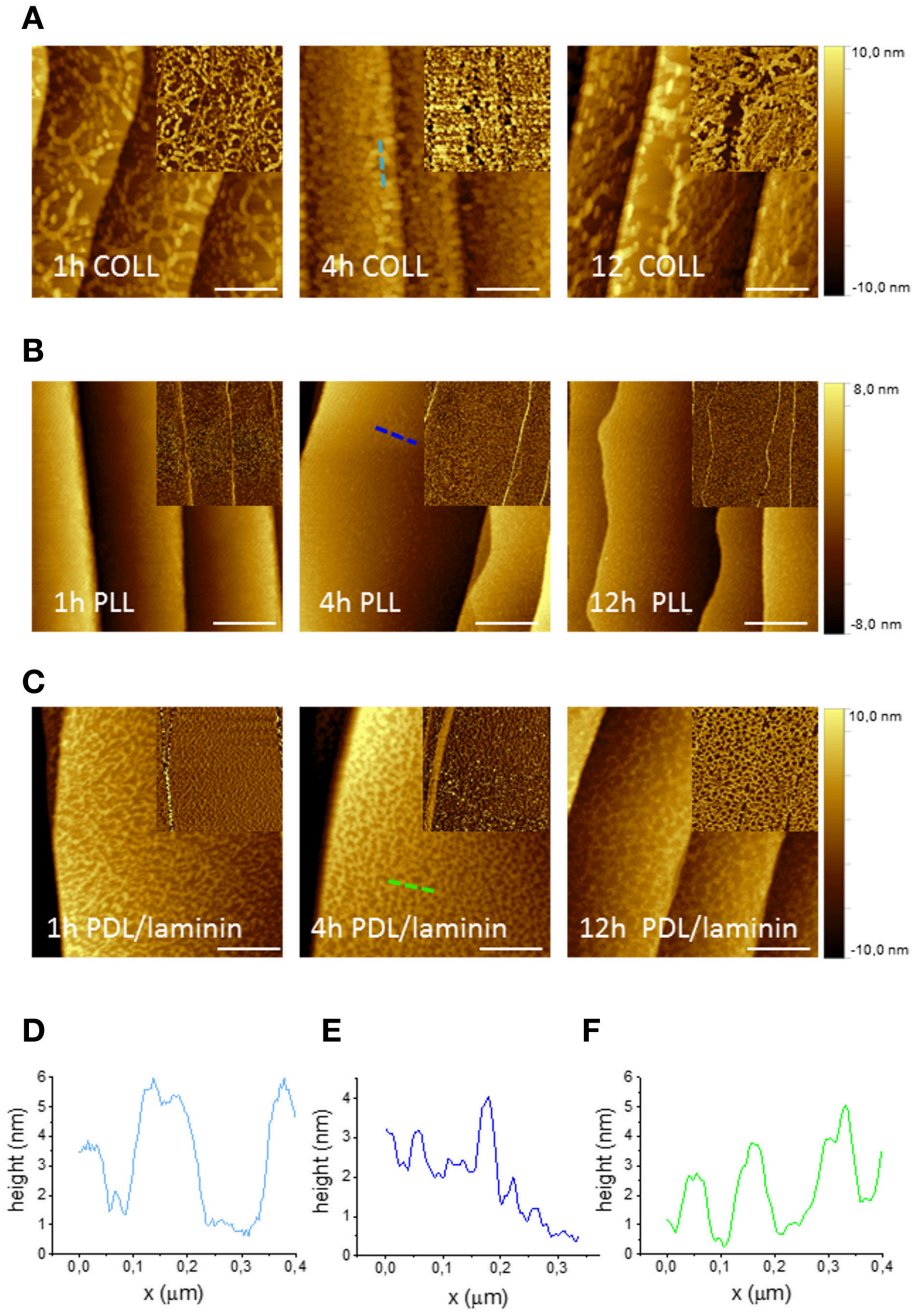
AFM micrographs of graphene with various polymeric coatings after different incubation times. AFM topography images of graphene after three different times of incubation (1, 4 and 12 h) with Collagen Type I coating (200 μg/ml in DI water) **(A)**, Poly-L-lysine (100 μg/ml in DI water) **(B)** and Poly-D-Lysine and Laminin coating (30 μg/ml PDL and 5 μg/ml laminin in PBS) **(C)** (scale bar: 500 nm). The insets show phase images of the same areas. **(D–F)** AFM height profile, along the dashed lines with corresponding color in the images, of graphene with each coating after 4 h-incubation.

NGF-induced neurite outgrowth of PC12 cells is favored by their adhesion on a substrate. This is typically achieved by coating the dish surfaces with polymers such as poly-L-lysine or biologically derived collagen (Greene and Tischler, 1982). We applied a water solution of both these coatings to all substrates adopted for our cultures and analyzed by AFM the quality and homogeneity of the coatings after different incubation times, i.e., 1, 4, and 12 h. Figures 1A,B show AFM phase and topography micrographs for the two different coatings and different incubation times on a graphene substrate. Clearly, the Poly-L-Lysine (PLL) coating presents better homogeneity with respect to Collagen Type I coating for which network-like aggregates can be detected (Figures 1D,E). On the other hand, PLL tends to form a homogeneous carpet of spots of 1–2 nm (no aggregates) independent from the incubation time. We also analyzed the same coatings on SiC, gold and glass surfaces. On SiC, PLL and Collagen presented analogous topographies (Figures S2a,b). Due to the higher surface roughness of gold and glass substrates (presenting rms roughnesses of about 1 nm comparable to the features of the polymeric layer), no conclusions about the quality of the coating could be drawn (Figures S3a,b). However, presence of the coating was confirmed by the variation in the hydrophilicity observed with contact angle measurements (Figure S3c). Hence, for the PC12 cells cultured in this work, a PLL coating with an incubation time of 4 h was adopted.

The same characterization was performed for the polymeric coatings typically suggested for DRG neurons, i.e., PBS solution of Poly-D-Lysine (PDL) alone and PDL with laminin. Figure 1C shows the AFM topography and phase images taken for PDL/laminin coated graphene substrates for the three different incubation times (i.e., 1, 4, and 12 h). Also in this case, after the coating, an increased roughness was observed for all time points and in particular the formation of a network-like structure was consistently observed (Figure 1F). PDL alone coating gave rise to a similar net (Figure S4b). In order to exclude the effect of PBS, we dissolved the same polymeric amount in DI water and after 4h incubation we observed similar structures (Figure S4a). To check if the different molecule arrangement of PLL and PDL on graphene was dependent on their concentration, we tested also a PDL coating solution in DI water with the same concentration used for PLL (100 μg/ml). We obtained structures similar to the ones observed for the lower PDL concentration (Figure S4c). On SiC no network formation was observed with or without laminin (Figures S2c,d). The stability of the coating was confirmed for all the probed incubation times. In this case, PDL with laminin coating (with an incubation time of 4 h) was selected to carry on the following DRG culture experiments in order to mimic the extracellular matrix.

Interestingly, the coating solutions distributed differently on graphene and SiC, despite their similar morphologies before the coating, with nanometric terraces and comparable roughness (Figures S5a,b). All polymeric coatings exhibited similar distributions on SiC, while there were significant differences between the coatings on graphene. The dissimilar arrangement of the coatings on the substrates can be reasonably ascribed to the different hydrophilicity of graphene and SiC (Oliveros et al., 2011). As shown by the contact angle measurements reported in Figure S5c, graphene is in any instance (pre and post-coating) more hydrophobic than SiC. The contact angle estimated for graphene was 95.8° ± 1.3° while it was 38.3° ± 7.2° for SiC, in agreement with literature (Coletti et al., 2007; Wang et al., 2009; Oliveros et al., 2011). SiC hydrophilicity likely facilitated (for all the various coatings adopted) a homogenous adhesion of molecules. The network-like structures often revealed by our analysis on graphene indicate that such pristine hydrophobic surfaces are less prone to be homogenously coated, an important aspect that should be considered in future works when studying cell cultures on graphene.

### Neurite outgrowth of PC12 cell on graphene

**Figure 2:**
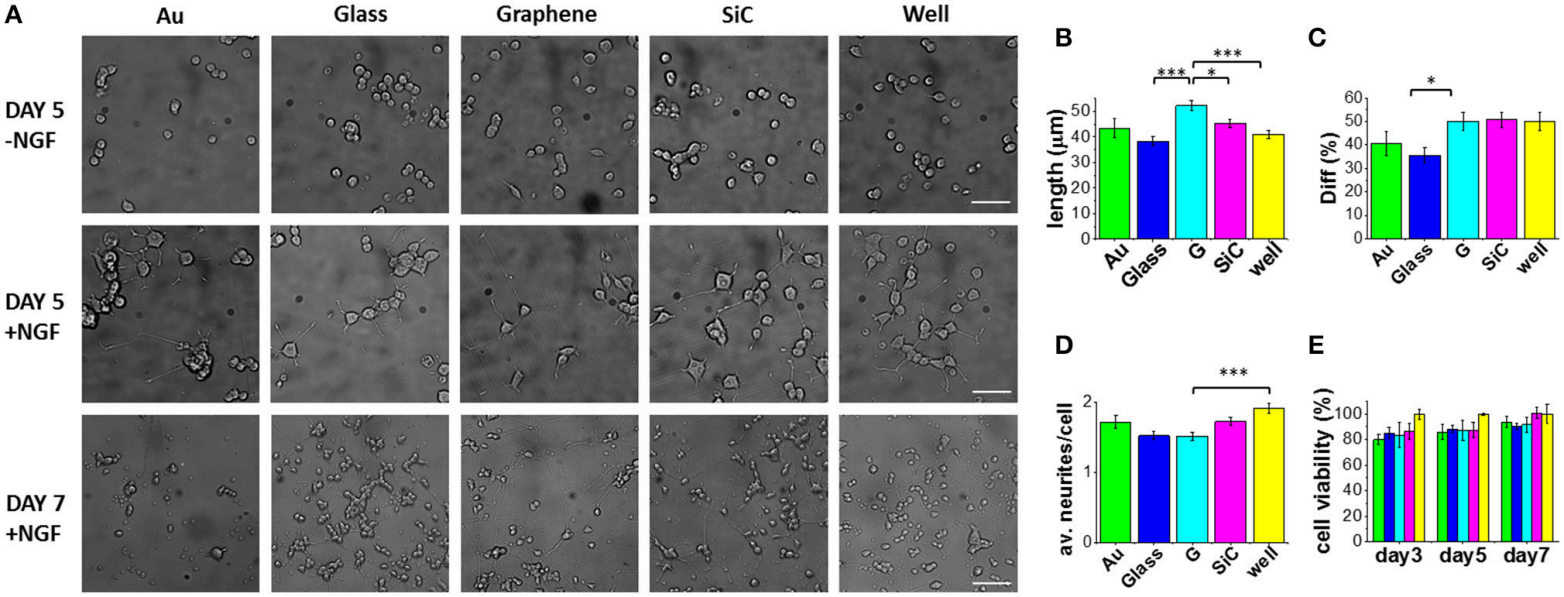
PC12 cells cultured on graphene and control substrates. **(A)** Typical optical microimages of PC12 cells grown on gold (Au), glass coverslip (Glass), graphene (G), SiC and polystyrene (well) coated with Poly-L-lysine (100 μg/ml in DI water), 4 h incubation) in the absence of NGF (first row, scale bar: 50 μm), PC12 cells differentiation at day 5 (second row, scale bar: 50 μm) and day 7 (third row, scale bar: 100 μm). Histograms show the quantification of **(B)** neurite length, **(C)** percentage of differentiation and **(D)** average number of neurites per cell after 5 days of NGF treatment of two independent experiments per substrate. For each substrate we analyzed at least 200 cells (nc) from selected fields (nf) (Au: nf = 17, nc = 203; Glass: nf = 33, nc = 1106; G: nf = 42, nc = 877; SiC: nf = 35, nc = 1004; well: nf = 37, nc = 724). **(E)** Cell viability after 3, 5, and 7 days tested by WST-8. The results are reported as % over the polystyrene control sample. Bars colored as in the other graphs. Data reported as mean ± SE. Nonparametric Kruskal–Wallis test was used for statistical significance, with ^*^p < 0.05, ^***^p < 0.001.

We first investigated the effect of graphene on PC12 cells. Figure 2A reports typical optical micrographs obtained for PC12 cells cultured at day 5 (in the presence and absence of NGF) and at day 7 (with NGF) on the different substrates. The analyses conducted at day 5 evidence that almost no differentiation took place in the absence of NGF, while a significant neurite outgrowth occurred on all substrates upon NGF treatment.

Selected morphometric parameters describing the differentiation process were quantified at day 5 and are reported in Figures 2B–D: the percentage of differentiated cells in the fields (Diff), the average number of neurites per cell (av. neurites/cell) and the length of the longest neurite per differentiated cell (length). This analysis showed that 50% of the cells on graphene differentiate with a mean neurite length of 52.3 μm (Figures 2B,C). Remarkably, the average length was significantly longer on graphene than on glass (^***^), well (^***^) and SiC (^*^) by 27, 22, and 13%, respectively. The percentage of differentiation on graphene was better than on glass (^*^), while the average number of neurites per cell was lower on graphene than on the control well (^***^). These results indicate that PC12 cells grow longer neurites on graphene, with a neuronal differentiation that is comparable to that obtained for the standard control wells. Differently from reference (Hong et al., 2014), we did not observe increased PC12 proliferation on graphene, which could be due to the effect of the FBS coating used in that study. Furthermore, we found that at day 7 living PC12 cells forming neurite networks were present on all the substrates. To better assess graphene cytocompatibility, the viability of undifferentiated PC12 cells was assessed after 3, 5, and 7 days of culture and no statistically significant differences were observed between graphene and the other substrates (Figure 2E). These data are in agreement with previous observations that graphene induces neurite sprouting and outgrowth of hippocampal neurons due to an overexpression of growth-associated protein-43 (GAP-43) (Li et al., 2011). Also, Lee et al. showed an induced neurite outgrowth of human neuroblastoma (SH-SY5Y) cells on graphene, probably mediated by focal adhesion kinase (FAK) and p38 mitogen-activated protein kinase (MAPK) cascades and upregulation of genes involved in neurogenesis (NFL, nestin and MAP2) (Lee et al., 2015). Both the studies excluded a neurogenic effect from substrate topography and wettability. Thus, we speculate that also for PC12 cells, graphene surface chemistry and electrical conductivity can specifically increase neurite length during differentiation.

### DRG primary neurons on graphene

**Figure 3:**
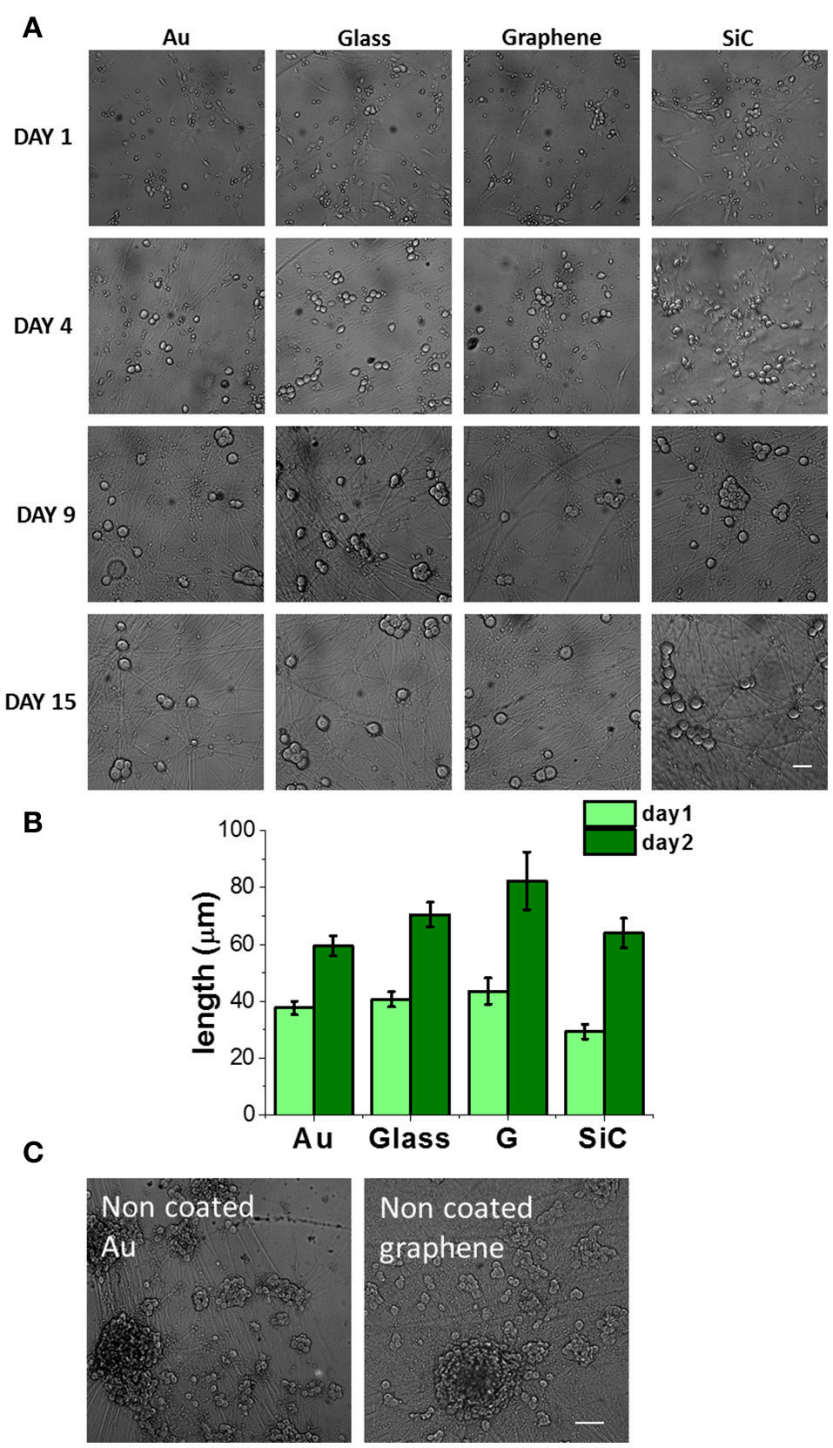
DRG neurons cultured on graphene and control substrates. **(A)** DRG neurons cultured on gold (Au), glass coverslip, graphene (G) and SiC coated with Poly-D-lysine and laminin (30 μg/ml PDL and 5 μg/ml laminin in PBS, 4 h incubation) at different days of culture. Scale bar: 50 μm. **(B)** Axon length quantification at 24 and 48 h after cell seeding. We analyzed nf fields for a total of nc cells for each substrate (day 1: Au, nf = 13, nc = 67, Glass: nf = 14, nc = 75; G: nf = 13, nc = 29; SiC: nf = 12, nc = 35; day 2: Au, nf = 16, nc = 89, Glass: nf = 13, nc = 100, G: nf = 12, nc = 34, SiC: nf = 11, nc = 37) and data are reported as mean ± SE. **(C)** DRG neurons on bare gold and graphene at day 10. Scale bar: 100 μm.

Next, we investigated the effect of graphene on primary neurons using dorsal root ganglion (DRG) cells while using the same controls adopted in the previous culture. As motivated in section Polymeric Coating of Epitaxial Graphene and Control Substrates, all the samples were coated with PDL/laminin. Figure 3A shows typical optical microscopy images obtained at 1, 4, 9, and 15 days of culture. Starting from day 4, we observed numerous processes and an increase in the cell body area (Figure S6) and in the neurite length (Figure 3A). Neurons were observed on all the substrates up to 17 days of culture. We observed that both at day 1 and day 2 the average axon length was higher on graphene than on the other substrates (Figure 3B). This observation confirms the trend reported for PC12, although in this case no statistical significance was retrieved. Axonal length was not quantified for longer culturing times due to the highly dense network forming after day 2 (see day 9 and 15 in Figure 3A).

Given that neuronal growth was previously reported also for non-coated graphene (Wang et al., 2011; Bendali et al., 2013; Sahni et al., 2013; Defterali et al., 2016; Fabbro et al., 2016; Veliev et al., 2016; Keshavan et al., 2017), we tested also the bare substrates to observe their effect on the neurons. Differently from non-coated glass, where they did not survive, DRG neurons could be nicely cultured on non-coated graphene and gold up to 17 days. On coated graphene neurons distributed homogeneously on the entire samples (Figure 3A and Figure S7a), while on uncoated graphene neurons formed small interconnected cell islets already after 24h from seeding (Figure S7a). After 2-3 days of culture, we observed neurites sprouted from the islet toward the substrate, and at longer times neurons formed cell bodies aggregates and neurite bundles (Figure 3C and Figures S7a,c), probably due to a reduced neural adhesion in the absence of coating, as previously observed for retinal ganglion cells (Bendali et al., 2013) or cortical neurons (Sahni et al., 2013). We rarely observed neurite bundles on coated graphene, while they were present on uncoated graphene already after 2 days of culture and they increased in size with time (Figure S7d). Cell body area was comparable with the one on coated graphene. Higher cell body area on uncoated graphene was observed starting from day 4, but the values did not differ significatively (Figure S7b).

In order to improve adhesion and neuron homogeneous distribution, the surface modification with an hydrophilic coating turned out to be useful (Li et al., 2011; Keshavan et al., 2017). Moreover, as previously suggested, the coating could mask the presence of surface inhomogeneity and defects that affect neural adhesion (Veliev et al., 2016).

Concerning material stability issues, it should be noted that graphene showed a good stability and remained intact during the entire culturing period, as revealed by Raman measurements after cell removal (Figure S8).

## Conclusion

This work provides novel data about the use of graphene as a substrate for peripheral neuron cultures. We chose to use graphene on SiC because, thanks to its high quality and cleanliness, it allowed us to examine the graphene effect on peripheral neurons with fewer concerns for contaminations and crystalline quality that may affect neuron adhesion (Veliev et al., 2016).

We use the PC12 cell line as a consolidated model for peripheral sympathetic neurons and show that such cells grow well on graphene with an increased neurite length (up to 27%) at 5 days of differentiation when compared to controls. Remarkably, graphene performs better than gold, which we used as conductive control. Culture of DRG neurons also shows a positive outcome on graphene: neurons survive both on bare and coated graphene until day 17, with a dense axon network that is comparable to the control substrates. In order to investigate graphene influence on axonal outgrowth, further studies are necessary, e.g., using compartmentalized chambers (Taylor et al., 2005). The obtained results confirm the potential of graphene as an active substrate in conduit devices for nerve guidance: it would allow the transmission of electrical signals between neurons and make external electrical stimulation feasible to enhance axon regeneration. While for many biomedical applications graphene-based materials with higher roughness might be desirable, in specific cases when high transparency and electrical conductivity are required, pristine highly crystalline graphene might be the ideal choice (Kuzum et al., 2014; Reina et al., 2017). It should be noted that flexibility is a requirement in neural regeneration that cannot be met by using graphene on SiC. To use graphene as neural interface other graphene production methods, such as chemical vapor deposition (CVD), could be more suitable.

## Author contributions

DC, LM, and CC conceptualized the study, DC performed the experiments. All the authors discussed the results. The manuscript was written through contribution of all authors.

### Conflict of interest statement

The authors declare that the research was conducted in the absence of any commercial or financial relationships that could be construed as a potential conflict of interest.
