# Supplementary material for: Peripheral Neuron Survival and Outgrowth on Graphene
Source: Front Neurosci. 2018 Jan 22;12:1. doi: 10.3389/fnins.2018.00001 (PMC5786521; doi:10.3389/fnins.2018.00001)
Supplement: Supplementary file 1 [file Image1.pdf]

Supplementary Material

Peripheral neuron survival and outgrowth on graphene

Domenica Convertino, Stefano Luin, Laura Marchetti*, Camilla Coletti*

*** Correspondence:** Corresponding Author: camilla.coletti@iit.it, laura.marchetti@iit.it

Graphene samples characterization

The samples were characterized by AFM (Fig. S1(a)) and Raman spectroscopy (Fig. S1(b)). An AFM+ from Anasys Instruments operated in tapping-mode was used to scan several areas. The analysis of the graphene surface topography shows large continuous terraces separated by steps. A micro-Raman spectroscope was used to map the characteristic graphene 2D peaks. The position and shape of the 2D (~2700 cm-1) peak, originated from a double resonance electron-phonon scattering process, give an indication of the doping and the number of graphene layers. In particular, the single Lorentzian fitting of the peak is characteristic of monolayer graphene, while for bilayer and trilayer graphene the 2D peak becomes broader and the fitting requires multiple Lorentzians. The energy of the peak, blue-shifted with respect to the case of pure undoped graphene, indicates a p-type doping, characteristic of a quasi-free standing monolayer graphene (QFMLG).

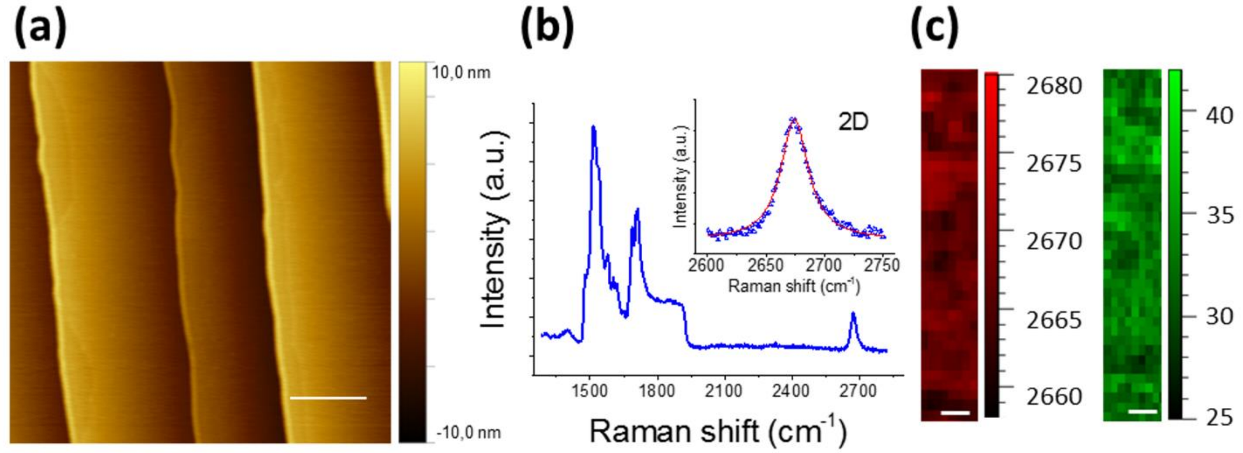

Figure S1. (a) Characteristic AFM topography of an intercalated graphene sample, showing atomically flat terraces separated by steps (scale bar: 400 nm). (b) Raman spectrum of an intercalated graphene sample, obtained using a 532 nm laser and a 50x objective lens. The insert shows the single Lorentzian fitting of the 2D peak, with a narrow FWHM of 28 cm-1. (c) 2D peak position (left) and FWHM (right) distribution in a large area (scale bar: 2 µm).

AFM Topography of SiC after three different incubation times with polymeric coatings

AFM analysis of SiC substrates after different polymeric coating show similar topographies with a homogeneous carpet of spots of few nanometers (Fig. S2(e)). The substrates were incubated with the following coating solutions at 37 °C for 1, 4 and 12 h: (a) PLL, 100 µg/ml Poly-L-lysine in water; (b) COLL, 200 µg/ml Collagene Type I in deionized (DI) water; (c) PDL, 30 µg/ml Poly-D-lysine in PBS; (d) PDL/laminin, 30 µg/ml PDL and 5 µg/ml laminin in PBS.

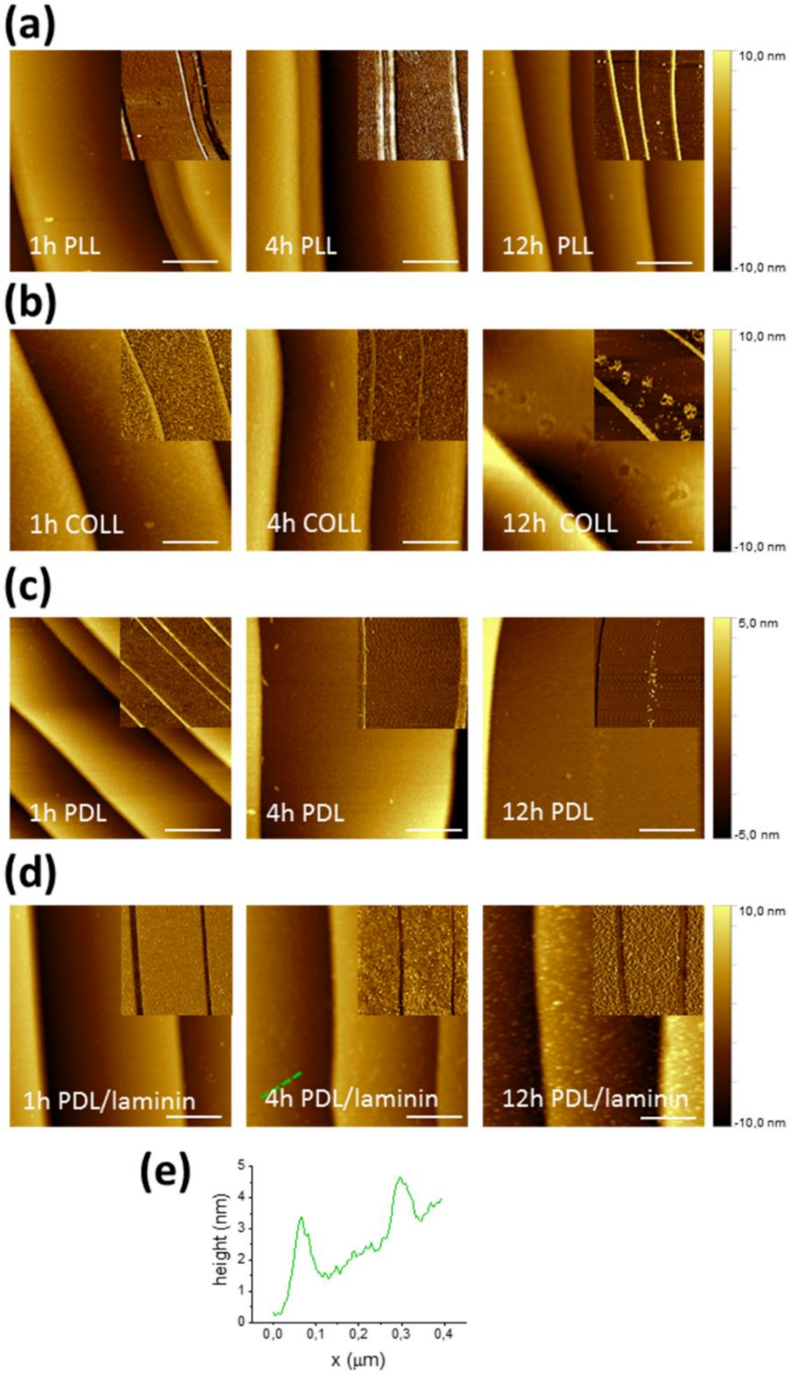

Figure S2. AFM topography images of SiC samples after three different times of incubation (1, 4 and 12 h) with a coating solution of: (a) PLL, (b) collagen, (c) PDL, (d) PDL/laminin (scale bar: 500 nm). The insets show phase images of the same areas, which are not sensitive to slow changes in height and improve identification of nanometric structures. (e) All the samples are coated with a homogeneous carpet of spots of few nanometers, as showed in the AFM line profile of a SiC sample after 4 h incubation with PDL/laminin.

Topography and hydrophilicity of gold and glass

Gold (Au) and glass show a relatively high surface roughness already before the coating, with a root-mean-square (rms) roughness comparable to the features of the polymeric layer. However, the variation in the hydrophilicity confirmed the presence of the coating, as shown by the contact angle measurements reported in figure S3(c). Non-coated gold was more hydrophobic than non-coated glass. The coatings had opposite effects on the substrates, increasing hydrophilicity for gold and increasing hydrophobicity for glass. Contact angles were measured using a CAM 101 contact angle meter, from KSV Instruments Ltd. (Finland) and estimated by measuring the angles between the baseline of the droplet and the tangent at the droplet boundary.

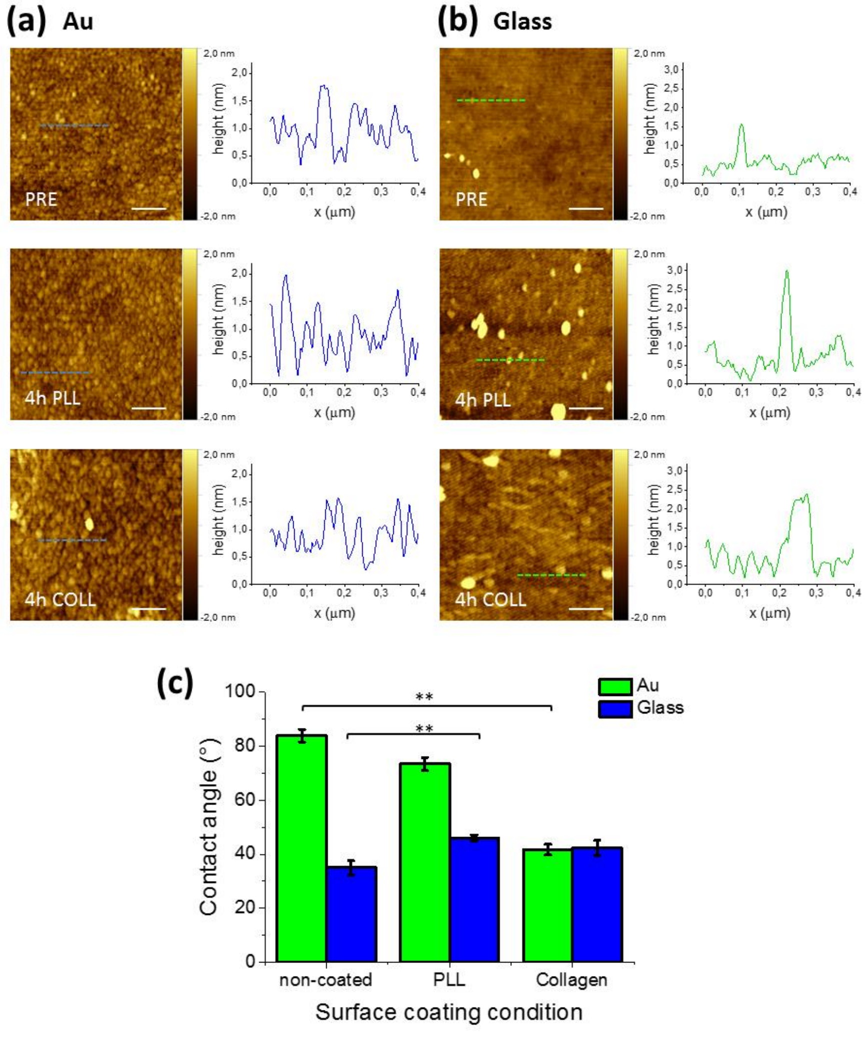

Figure S3. AFM topography and roughness profiles of gold (a, Au) and nitric-acid-treated glass (b, Glass) before protein coating and after 4h incubation with Poly-L-lysine (4h PLL) and Collagen Type I (4h COLL) (scale bar: 200 nm). Both the surfaces revealed an initial roughness comparable to the one after any coating, preventing the recognition of nanometric details of the coatings. (c) Contact angle measurements of Au and Glass before protein coating and after 4h incubation with Poly-L-lysine (PLL) and Collagen Type I (Collagen). All measurements were made using DI water as a probe liquid. Values are the mean ± standard deviation for 3 samples.

AFM Topography of graphene after different coating solutions

We tested the effect of different PDL coating solutions on graphene substrates. We obtained similar network-like structures for all the conditions: (a) PBS and water solution of PDL/laminin (30 µg/ml PDL and 5 µg/ml laminin), (b) PBS solution of PDL alone (30 µg/ml) and (c) water solution of PDL at higher concentration (100 µg/ml).

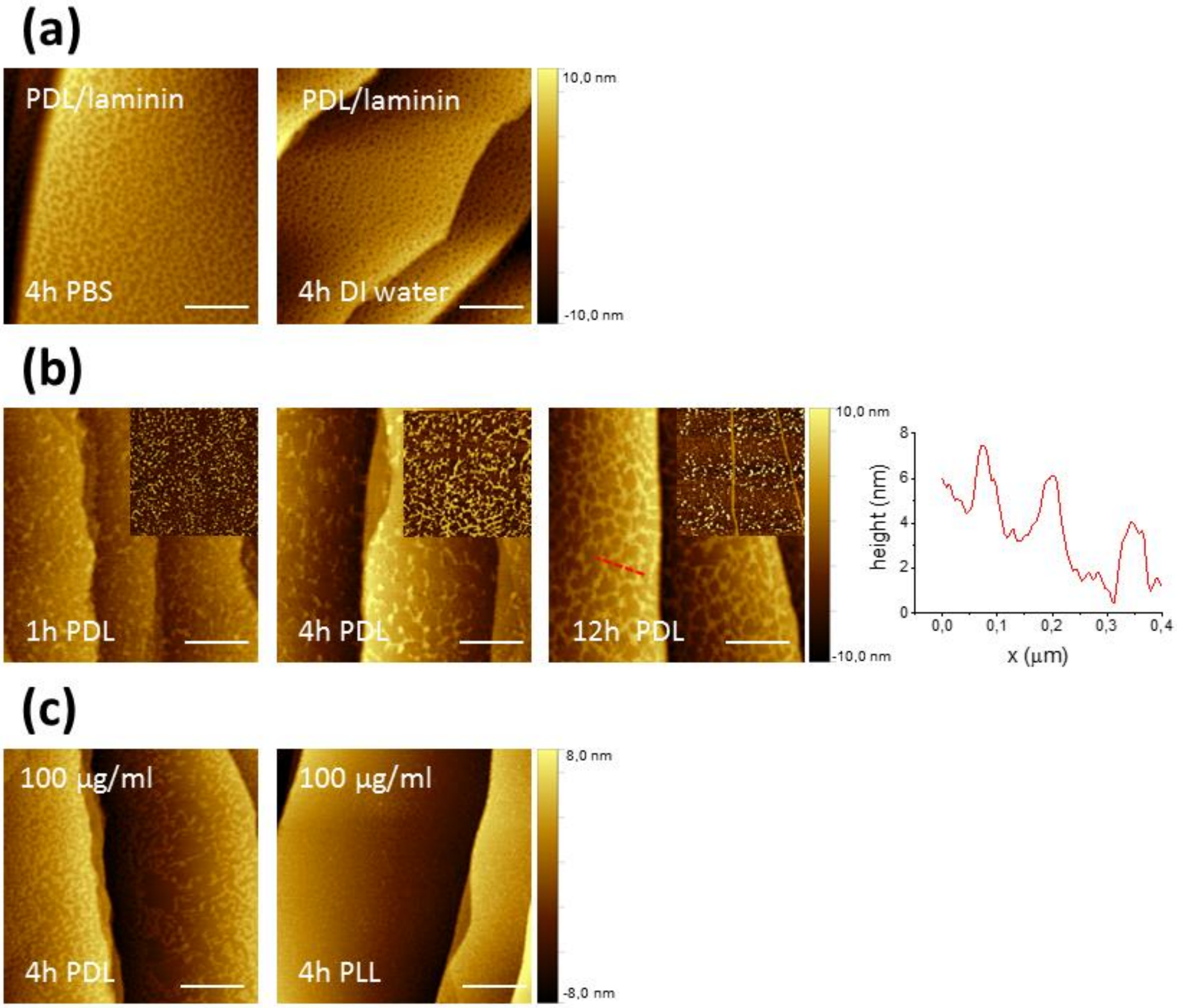

Figure S4. (a) AFM topography of graphene samples coated with PDL/laminin dispersed in DI water and PBS after 4h incubation show similar net structures. This implies that the net morphology is independent from the salts in the PBS solution. (b) AFM topography images with a characteristic line profiles of graphene after three different times of incubation (1, 4 and 12 h) with Poly-D-Lysine (PDL) coating. (c) AFM topography of graphene samples coated with PDL and PLL in water at the same concentration (100 µg/ml) after 4h incubation have different arrangements, showing that the different morphology is not dependent on the concentration but probably on the molecular weight of the two polypeptides (scale bar: 500 nm).

Topography and hydrophilicity of SiC and graphene

AFM analyses of graphene and SiC substrates evidence a similar surface topography with a comparable rms roughness (Fig. S5(a) and (b)). However, the two substrates present distinct differences in hydrophilicity, with SiC significantly more hydrophilic than graphene, as shown by contact angle measurements (Fig. S5(c)).

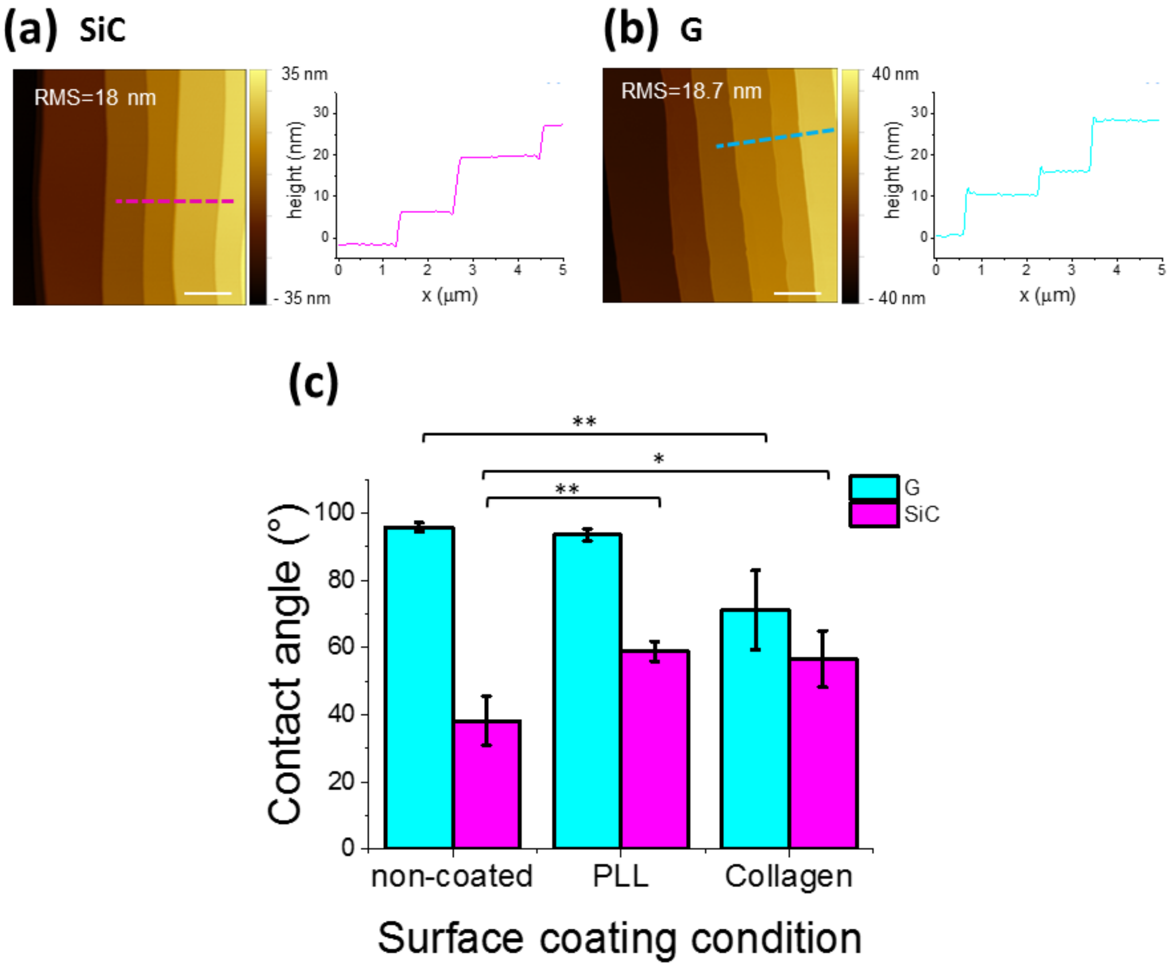

Figure S5. AFM topography of SiC (a) and bare graphene, G (b) samples, with characteristic line profiles across the terraces. Scale bar: 2µm. (c) Contact angle measurements of silicon carbide (SiC) and graphene (G) before protein coating and after 4h incubation with Poly-L-lysine (PLL) and Collagen Type I (Collagen). All measurements were made using DI water as a probe liquid. Values are the mean ± standard deviation for 3 samples. Non-coated graphene was more hydrophobic than non-coated SiC.

Dorsal root ganglion cell body area

Cell bodies show an increased area with culture time. To estimate the body area, cell bodies were approximated to an oval shape and relative areas were evaluated using ImageJ.

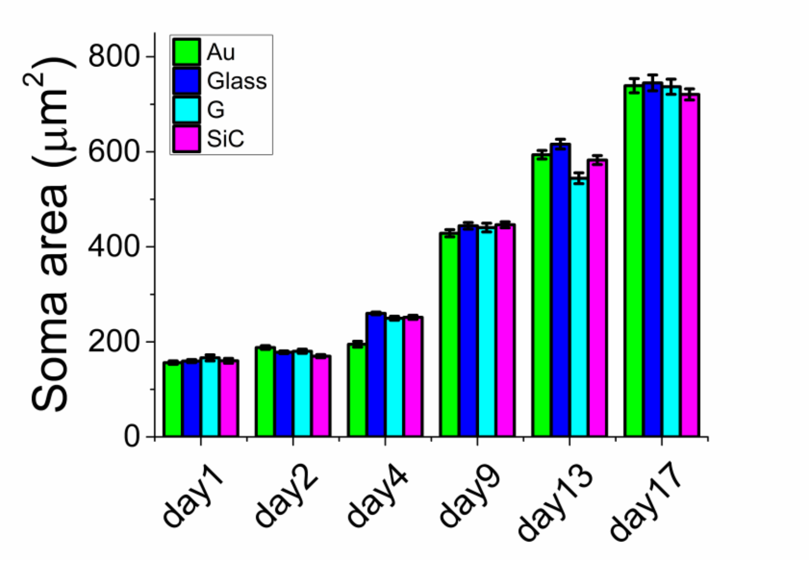

Figure S6. Increase of the cell body area with time in dorsal root ganglion (DRG) cells. For cell soma analyses more than 100 cells per sample were analysed.

DRG neurons on coated and uncoated graphene

DRG neurons show a uniform distribution on coated graphene and interconnected cell islets on uncoated graphene, already after 24h from seeding (Fig. S7(a)). With time, neurons covered homogeneously the coated samples, while cells islets with significant axonal fasciculation were observed on uncoated graphene (Fig S7(c) and (d)). Cell body area, calculated for the isolated cells, was comparable with the one on coated graphene as shown in Fig S7(c)

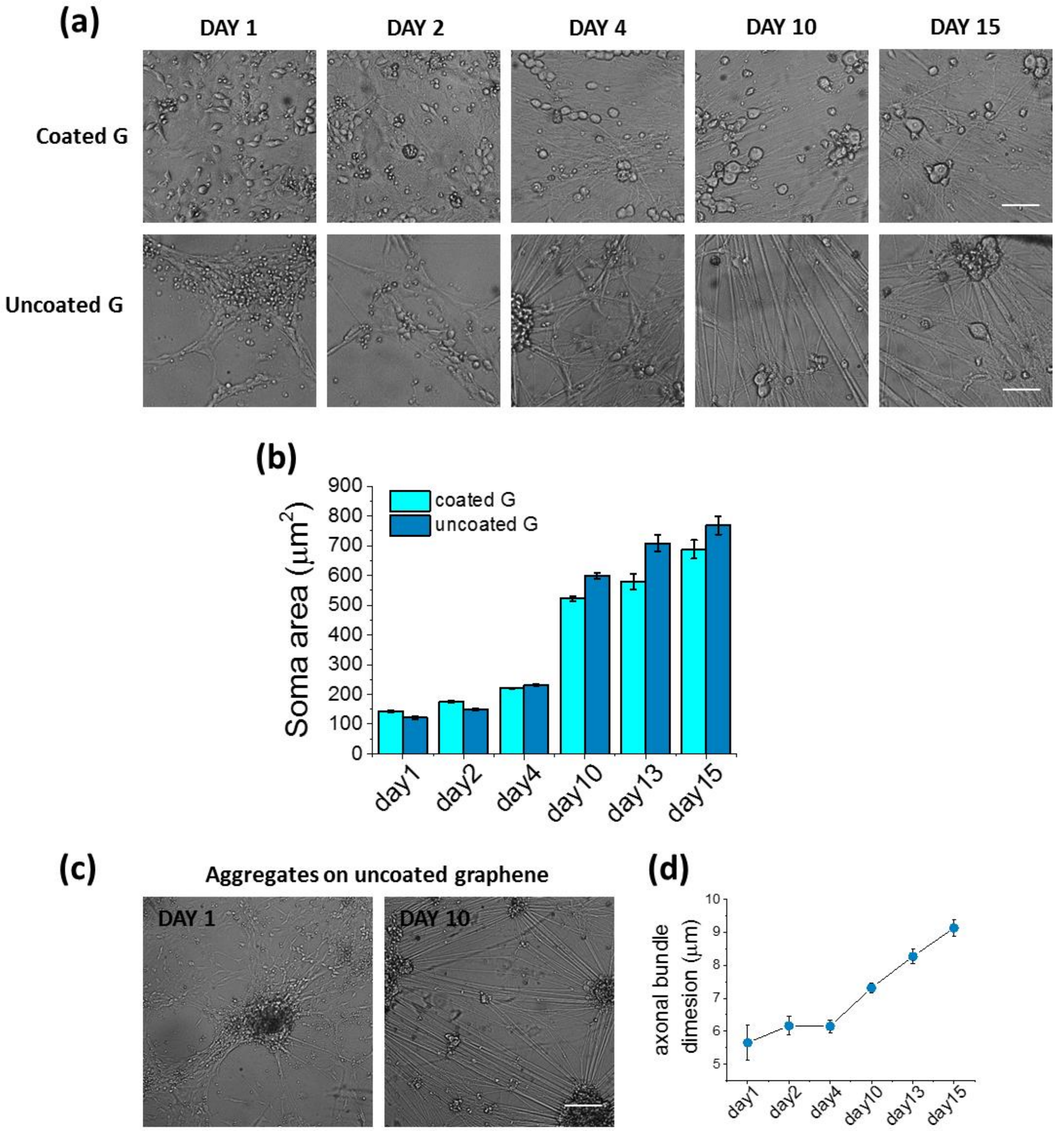

Figure S7. (a) DRG neurons cultured on coated and uncoated graphene at different culture days. Scale bar: 50 µm. (b) Increase of the cell body area with time in dorsal root ganglion (DRG) cells on coated and uncoated graphene. (c) Cell bodies aggregates and neurite bundles on uncoated graphene at different days of culture. Scale bar: 100 µm. (d) Quantification of axonal bundles dimension on uncoated graphene. The diameter of the axonal bundles was evaluated using ImageJ. Data are reported as mean ± SE.

Raman characterization after cell culture

Graphene remains of high quality after cell culture, as reported in Fig S8. The maps reveal that the 2D peak and FWHM are very homogeneous across the whole area and the values resemble those measured before the cell culture, with a narrow 2D peak of ~30 cm-1 centered at ~2670 cm-1.

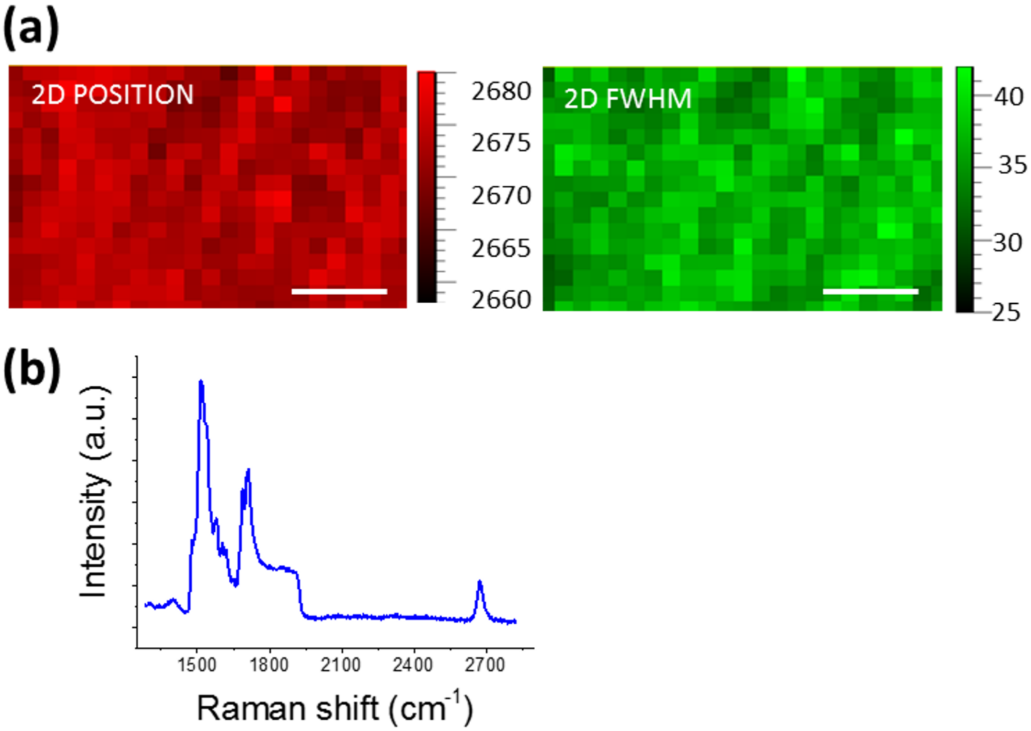

Figure S8. Raman characterization with 532 nm laser of a graphene sample after cell culture validates the full coverage of graphene; Raman was excited with a 532-nm laser. (a) 2D peak position and FWHM distribution in a large area (scale bar: 5 µm). (b) Characteristic Raman spectrum.
